# Retraction statement: The PI3K/AKT axis modulates AATF activity in Wilms' tumor cells

**DOI:** 10.1002/2211-5463.13514

**Published:** 2022-11-15

https://doi.org/10.1002/2211-5463.12500

The above article published online on 04 August 2018 in Wiley Online Library (wileyonlinelibrary.com), has been retracted by agreement between the Editor‐in‐Chief, John Wiley and Sons Ltd and the authors. The retraction has been agreed following an investigation based on allegations raised by a third party, which revealed that some images in this article are inappropriate duplications of images from a previously published article [1]. Thus, the editors consider the conclusions of this manuscript substantially compromised.
